# Detection of seroconversion to West Nile virus, Usutu virus and Sindbis virus in UK sentinel chickens

**DOI:** 10.1186/1743-422X-3-71

**Published:** 2006-09-04

**Authors:** Alan Buckley, Alistair Dawson, Ernest A Gould

**Affiliations:** 1 CEH Oxford, Mansfield Road, Oxford OX1 3SR, UK; 2 CEH Monks Wood, Abbots Ripton, Huntingdon, Cambridgeshire PE28 2LS, UK

## Abstract

We previously reported evidence of West Nile virus (WNV) circulation in UK birds, probably introduced by migratory birds from overseas. We now demonstrate WNV-specific seroconversion in sentinel chickens raised on an English farm. Maternal neutralizing antibodies to WNV in hatchlings declined within three weeks. During the following months, healthy chickens developed WNV neutralizing antibodies that were confirmed by immunoblotting and indirect immunofluorescence tests using WNV antigens. The proportion of seropositive chickens was higher for WNV than for Usutu virus or Sindbis virus. Attempts to isolate infectious virus or to detect viral RNA in the sera, failed.

## Background

West Nile virus (WNV) and Usutu virus (USUV) are antigenically closely related mosquito-borne members of the genus *Flavivirus*. Sindbis virus (SINV) is an unrelated mosquito-borne member of the genus *Alphavirus*. These **ar**thropod-**bo**rne viruses (**arbo**viruses), and many others, are known to circulate globally as pathogens amongst birds and mammalian species [1-4]. During their natural life cycles, they infect ornithophilic *Culex *spp. mosquitoes that replicate and transmit the viruses to birds and/or mammals when they feed on them. Fatal encephalitic infections of avian species have been recorded for WNV in North America [5-7], and Israel, [8] and for USUV in Austria [9]. Nevertheless, many healthy avian species have antibodies to these viruses, demonstrating that they are not necessarily pathogenic for all species they infect. On the other hand, WNV and SINV are known human pathogens and have been shown to be pathogenic for a very wide range of other mammalian species both in North America and in the Old World [10]. Previous serological studies on sera collected from UK resident and migratory birds demonstrated the presence of WNV-specific neutralizing antibodies and also small fragments of RNA with sequence corresponding to WNV. We also previously demonstrated the presence of WNV-reactive envelope and non-structural protein (NS1) antibodies by western blot analysis and by indirect immunofluorescence (IF) tests using WNV-infected tissue culture cells as the substrate for the IF tests. The presence of antibodies to NS1 protein inferred that the virus had replicated in the birds since non-structural proteins are only produced in infected cells after virus replication, ie they would not be present in an introduced virus. However, in view of the need for additional proof of the presence of WNV circulating amongst birds in the UK, albeit apparently harmlessly, we have looked for evidence of seroconversion to WNV, USUV and SINV in sentinel chickens.

## Results and discussion

### Plaque reduction neutralization tests on sentinel chicken sera

**Figure 1:**
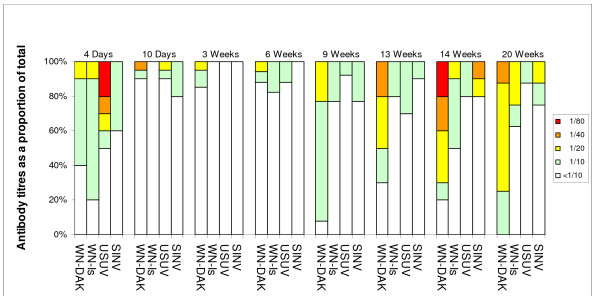
Neutralization results (PRNT_50_) obtained for all sentinel chicken sera tested against each of the four viruses and grouped according to age at time of sampling. All chicken sera were coded and all tests were carried out on these coded sera. The codes were revealed only after the results had been presented. The percentages of positive sera recorded at each antibody dilution are shown using a colour scheme; White <1/10; Green 1/10; Yellow 1/20; Orange 1/40; Red 1/80.

All sera were tested for the presence of virus-specific neutralizing antibodies by plaque reduction neutralization tests (PRNT_50_) against two strains of WNV, a strain isolated from Israel (WN-Is) and a highly neutralization-sensitive strain isolated in the Central African Republic (WN-DAK). For these tests the sera were diluted in twofold steps from 1/10 dilution, the minimum possible, owing to the limited volume of serum. The World Health Organization (WHO) standard method based on 50% plaque reduction was employed to detect positive virus-neutralizing sera. Following the WHO recommendations, the highest dilution of serum that produced 50% reduction of plaque numbers (estimated 50 plaques per dish in control dishes) was taken as the endpoint for individual sera. In addition we also included USUV and SINV in this analysis because it extended the range of viruses analysed and also served as a form of internal control for virus-specificity. The results of plaque reduction neutralization tests (PRNT_50_) on the individual sera are presented in Fig 1. The inclusion of two strains of WNV maximized the data as we have previously demonstrated differences in sensitivity to neutralization of virus infectivity between different strains of WNV [3]. As shown in Fig. 1, the sera from 6/10 and 8/10 of the four-day old chicks neutralized WNV-DAK and WN-Is respectively, presumably reflecting the presence of maternal antibody in the hatched chicks. For USUV, 5/10 newly hatched chick sera contained detectable neutralizing antibody but they were not necessarily the same chicks that produced antibody against WNV, demonstrating the specificity of the neutralization test. However, by the time the chicks were 10 days old, the proportion of maternally derived neutralization positive sera against the two strains of WNV and against USUV had dropped to 2/20, 0/20 and 2/20 respectively and at days 21 and 46 the figures remained low, ie 3/20, 0/20 and 0/20 at day 21, but by day 46 the figures showed evidence of increasing, ie 1/10, 3/10 and 2/10. In the case of SINV, 4/10 four-day old chicks were positive. This figure then dropped to 4/20 ten-day old chicks and 0/20 chicks by day 21 and was still zero at day 46. From this time onwards, the proportion of WNV positive sera increased noticeably, until by October 8/8, and 7/8 of the sera were positive for WN-DAK and WN-Is respectively. In many cases the titres of these sera were noticeably higher than those recorded in previous months. In contrast, the proportions of seropositive chickens for USUV and SINV remained lower than those observed for WNV, once again demonstrating that the PRNT can discriminate between WNV and USUV. It is important to note that the major increases in WNV-antibody positive sera were detected in samples collected from late July to the end of September, regardless of the date of hatching of the chicks. Moreover, the results in Fig. 1 emphasise the importance of using a highly neutralization sensitive strain of virus, in this case the WN-DAK strain.

Interestingly, thirteen chicks sampled at 9 weeks post-hatching had been kept indoors for the entire period since hatching. Nevertheless, specific antibody responses to WNV in particular were detected in these chicks, viz., 12/13, and 6/13, for WNV-DAK and WNV-Is respectively and 1/13, and 3/13 for USUV and SINV respectively. However, whilst these chicks had been kept indoors, the airflows to their rooms came directly from the outside without isolation by filtration.

### Western blot analysis

**Figure 2:**
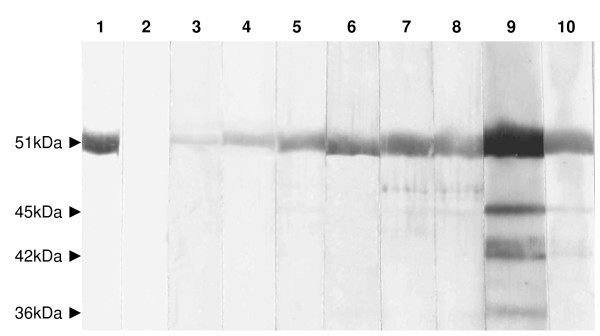
Western blot using gradient-purified West Nile virus antigen and sera (diluted 1/100) from sentinel chickens. Tracks 1 and 2, hyperimmune mouse serum prepared against WNV (positive control) or SINV (negative control) respectively; tracks 3 to 10 pairs of chicken sera that produced neutralization titres of 1/10, 1/20, 1/40, and 1/80 respectively.

Western blot analysis of sera was performed, to confirm the presence of antibodies to viral envelope glycoprotein, 7% w/v polyethylene-glycol-precipitated virus was purified by centrifugation at 100,000 g on a continuous sucrose gradient (15% to 60% w/w sucrose in Tris buffer at pH 7.4). Serial fractions were collected from the sucrose gradient and subjected to immunoblotting using a high titre hyperimmune mouse antiserum prepared against WNV. Whilst many fractions contained large quantities of the recognized structural WNV proteins, the fraction collected from the 60% sucrose cushion produced a very strong and relatively clean band at 51kDa on the western blot, corresponding to the viral envelope (E) protein of WNV as confirmed (data not shown but equivalent appearance to track 1 of Fig 2) using an E protein-specific monoclonal antibody (MAb) designated MAb 528 [11]. WNV-neutralization positive chicken sera were tested for the presence of E protein-specific antibodies using the gradient-purified fraction obtained from the 60% sucrose cushion (Fig. 2). As can be seen, tracks 3 to 10 produced increasingly intense labelling of the E protein when tested at a 1/100 dilution. These tracks corresponded to sera with neutralization titres of 1/10 (tracks 3 and 4), 1/20 (tracks 5 and 6), 1/40 (tracks 7 and 8), and 1/80 (tracks 9 and 10). Track 1 contained a positive control hyperimmune mouse antiserum against WNV and Track 2 contained a negative control hyperimmune antiserum raised against SINV (both positive and negative control sera were diluted 1/500). Chicken sera that failed to neutralize WNV were negative in immunoblots when tested at 1/100 dilution. The bands at 45, 42 and 36 kDa in track 9 of Fig 2 probably correspond to breakdown products of the E protein. They were only detected by chicken sera with the highest neutralization titres (tracks 9 and 10 of Fig 2). It is also important to note that separate immunoblots that employed non-purified WNV-infected cell lysates as substrate, and the 1/40 or 1/80 neutralization positive chicken sera, performed exactly as published previously [3], i.e., these neutralization-positive sera highlighted the viral E, NS1, NS3 and NS5 proteins, confirming that WNV must have replicated in the chickens to elicit immune responses against the non-structural viral proteins.

### Indirect immunofluorescence tests

**Figure 3:**
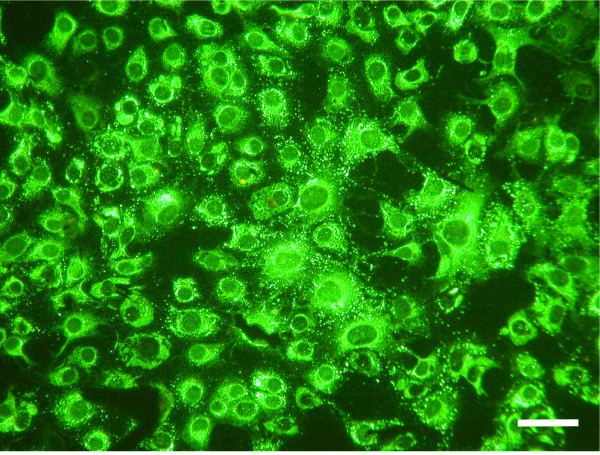
Indirect immunofluorescence microscopy performed on chicken sera with a neutralization titre of 1/80 (diluted 1:1000 in PBS) on West Nile virus infected Vero cells (Bar 50μm).

Indirect immunofluorescence microscopy was carried out using a PRNT-positive chicken serum (track 9 from Fig. 2) diluted 1/100 in PBS as described previously [3]. Vero cells infected with WNV for 48 hours at 37°C were washed in PBS and fixed in acetone. The diluted chicken serum was allowed to react with the acetone-fixed infected cells for 1 hour at 37°C before the cells were washed in PBS. Fluorescein-conjugated anti-chicken serum diluted 1/400 in PBS was used to identify the fluorescent infected cells. Fig. 3 illustrates the typical appearance of groups of WNV-infected fluorescent cells produced by PRNT-positive chicken antisera. In Fig. 3, non-infected cells are not stained by the antibody. Moreover, PRNT-negative control sera produced no fluorescence (not shown).

### Attempts to isolate infectious virus from seropositive chickens

The sera from 46-day old, and older chickens (a total of 46 sera) and five 10% brain suspensions harvested from chickens that were seropositive in a WNV-PRNT-analysis were inoculated directly onto monolayers of SW13 cells which were then incubated at 37°C for 14 days. The supernatant medium from each sample was then inoculated onto fresh monolayers of SW13 cells and these were incubated for a further 14 days. Each monolayer from the first inoculation and subsequently each monolayer from the second inoculation was tested for the presence of flavivirus antigens using a flavivirus pan-specific monoclonal antibody (MAb 813), followed by fluorescein-conjugated mouse antiglobulin, as described previously [11]. Although some monolayers deteriorated during the incubation period, suggesting that cytopathic effects (cpe) were developing, we were unable to demonstrate the presence of an infectious flavivirus in any of the tested samples either by indirect immunofluorescence microscopy using flavivirus-group-reactive MAb 813 or by RT-PCR using flavivirus-group-reactive primers [12]. Moreover, the mild cpe that was observed in some cultures was not observed during subsequent passage of harvested material, ruling out the possibility of a different cytopathic arbovirus being isolated.

Although it was not possible to obtain sequential samples of serum from each animal the PRNT studies with groups of newly hatched, juvenile and young adult chickens produced evidence that these animals had been exposed either to infectious WNV or a very closely related virus during the summer of 2004. The supplementary positive results obtained by immunoblotting and immunofluorescence microscopy also support this conclusion by demonstrating specific immune responses against the WNV envelope protein. Many of the newly hatched chicks had antibodies that neutralized WNV and to a lesser extent USUV and SINV. It is well known that maternal antibodies are concentrated in the fertile egg and that the quantity of these antibodies declines rapidly in the newly hatched chick [13]. Our PRNT results are totally consistent with these known observations and they demonstrate that WNV, USUV and SINV (at least), or closely related viruses, must have circulated on the farm in the previous year. The decline in antibody prevalence during the first few weeks after hatching is also consistent with the idea that WNV is unlikely to have been circulating significantly during the first three or four months of the year, i.e. late winter and early spring. The detection of a significant increase in the numbers of serologically positive chickens from July onwards can probably be explained most appropriately as due to this being the time immediately after the arrival of migratory birds from Africa, Europe and the Middle East and also being the warmest time of the year when mosquitoes would be relatively active and therefore capable of transmitting arboviruses, even in England. Some chickens seroconverted even though they had been kept indoors for most of their lives. However, the ventilation system for the building in which they were housed is positive and not filtered inwards, moreover, adjoining rooms contained wild birds, inferring that the chickens could have been exposed to aerosols containing virus. In addition to virus transmission by blood transfusion and organ transplantation, there is now compelling evidence that arboviruses such as WNV may be transmitted between vertebrates using a variety of mechanisms other than direct transmission by arthropods. These include the aerosol and faecal/oral routes, transmission via direct physical contact or maternal milk, and through contaminated water. It is also clear that WNV can persist in vertebrate hosts for months if not years without inducing obvious clinical symptoms [5,14-21]. It seems likely that these properties provide WNV with the tools to circulate silently in many regions of the world and this may explain our observations of seroconversion in sentinel chickens in the UK. It is also important to emphasize that similar studies using sera from sequentially bled sentinel chickens in Italy, known to circulate WNV but with no associated disease, have been carried out and will report similar findings to those reported herein (manuscript submitted for publication).

Our observations support and extend the findings of others that although mosquitoes are important vectors in disease transmission, other modes of transmission and persistence may also be important in the transmission and circulation of WNV and other arboviruses. We now need to understand why in most cases, WNV can disperse very successfully without causing overt disease but in other situations it can cause significant epidemic outbreaks involving substantial morbidity and mortality.

## Materials and methods

### Sentinel chickens

Three groups of chickens were hatched in early April, mid May and mid June 2004 respectively on a farm in Cambridgeshire and reared outdoors. Individual sera were collected from birds at various ages from 4 days to 20 weeks. The last samples (20 weeks) were collected at the end of October 2004, when outdoor temperatures had dropped sufficiently to reduce insect-biting activity in the UK to relatively low levels. Groups of these animals were monitored periodically for the presence, in the sera, of neutralizing antibodies to WNV, USUV and SINV. For obvious technical reasons, only very small quantities of serum were obtainable from the very young chicks, limiting the scope of their investigation. Another group of chickens was hatched and reared indoors, and serum samples collected at 9 weeks of age.

### Plaque reduction neutralization tests

These tests were carried out as described previously [3] and are based on the WHO standard method. Briefly, each heat-inactivated (56°C for 30 minutes) serum sample was diluted serially in twofold stages. These were mixed in equal volume with 50 plaque-forming units of either WNV-Is, WNV-DAK, USUV or SINV. The mixtures were incubated overnight at 4°C. Each mixture was then placed on a monolayer of SW13 cells in 24-well Petri-plates and incubated for 60 mins at room temperature. 1 ml of overlay medium (RPMI-1640 with Hepes buffer, 1% foetal bovine serum, penicillin, streptomycin and 1% SeaPlaque Agarose) was added to each well and allowed to set at room temperature, then the plates were incubated at 37°C until plaques were identifiable in control wells. The monolayers were fixed in 10% formol-saline and stained with 0.1% naphthalene black stain. Serum neutralization titres were estimated as the highest dilution causing at least 50% reduction of plaque numbers. Titres less than 1/10 were considered to be negative.

### Purification of WNV

The supernatant medium collected from 10 × 175 cm^2 ^plastic tissue culture bottles was clarified by centrifugation at 5000 g for 30 mins and the virus was then precipitated from this clarified medium by the addition of 7% polyethylene glycol and 0.4 M NaCl. After stirring overnight at 4°C, the virus was sedimented by centrifugation at 5000 g for 1 hour. The pellets were resuspended in PBS and layered onto 15–60% (w/w) sucrose gradients prepared in Tris-EDTA buffer pH7.4. The gradients were spun at 90,000 g for 3 hours and the tube was then fractionated by upward displacement. Each fraction was tested for the presence of viral antigens by western blotting (see below). The sample in the 60% sucrose fraction produced a very distinct band of viral envelope (E) protein as deduced using a monoclonal antibody known to bind to WNV-E protein (see Results).

### Western blotting

Gradient-purified West Nile virus antigen and sera (diluted 1/100) from sentinel chickens were used for the analysis. The virus proteins were separated by10% polyacrylamide gel electrophoresis under reducing conditions until the dye front had run off the bottom of the gel. A Biorad semi-dry blotter was used to transfer the protein bands from the gel onto the Hybond-P PVDF transfer membrane. After transfer the membrane was blocked in 5% milk powder (in TBS and 0.05% Tween 20) for 1 hour at room temperature. The blot was then cut into identical strips (approximately 6 mm wide) which were individually treated with a chicken serum diluted 1/100 to test for antibodies to WNV. The strips were washed in TBS/Tween 20 three times before addition of 1:20,000 dilution of Rabbit anti Chicken conjugated with alkaline phosphatase (Sigma) for 1 hour at room temperature. The strips were washed three times in TBS/Tween 20 then once in 0.1 M Tris pH9.6 before addition of the BCIP/NBT liquid substrate system (Sigma).

### Indirect immunofluorescence microscopy

This was performed on chicken sera (diluted 1:100 or 1:1000 in PBS). Each diluted serum was added to acetone-fixed WNV-infected Vero cells on glass coverslips. After incubation for 1 hour at 37°C the cells were washed in warm PBS for 30 minutes. Rabbit anti-chicken FITC (Sigma) diluted to 1:400 was then added and after incubation for 1 hour at 37°C, the coverslips were washed in warm PBS and water before mounting in DABCO/Glycerol/PBS pH8.6, on microscope slides. Each monolayer was examined for virus-specific immunofluorescence under a UV light microscope.

## Competing interests

The author(s) declare that they have no competing interests.

## Authors' contributions

AB carried out all the immunoassays and data processing and helped draft the manuscript, AD designed, set up and carried out the sentinel study, EAG conceived and co-ordinated the study, supervised the research and drafted the manuscript. All authors have read and approved the submitted manuscript.
